# The competition–dispersal trade‐off exists in forbs but not in graminoids: A case study from multispecies alpine grassland communities

**DOI:** 10.1002/ece3.4856

**Published:** 2019-01-11

**Authors:** Xiaolong Zhou, Chengzhi Li, Honglin Li, Qingdong Shi

**Affiliations:** ^1^ Institute of Arid Ecology and Environment Xinjiang University Urumqi China; ^2^ Key Laboratory of Oasis Ecology Ministry Education Xinjiang University Urumqi China; ^3^ State Key Laboratory of Plateau Ecology and Agriculture Qinghai University Xinjiang China

**Keywords:** alpine grassland, competitive ability, dispersal ability, functional trait, trade‐off

## Abstract

Much theoretical evidence has demonstrated that a trade‐off between competitive and dispersal ability plays an important role in facilitating species coexistence. However, experimental evidence from natural communities is still rare. Here, we tested the competition–dispersal trade‐off hypothesis in an alpine grassland in the Tianshan Mountains, Xinjiang, China, by quantifying competitive and dispersal ability using a combination of 4 plant traits (seed mass, ramet mass, height, and dispersal mode). Our results show that the competition–dispersal trade‐off exists in the alpine grassland community and that this pattern was primarily demonstrated by forbs. The results suggest that most forb species are constrained to be either good competitors or good dispersers but not both, while there was no significant trade‐off between competitive and dispersal ability for most graminoids. This might occur because graminoids undergo clonal reproduction, which allows them to find more benign microenvironments, forage for nutrients across a large area and store resources in clonal structures, and they are thus not strictly limited by the particular resources at our study site. To the best of our knowledge, this is the first time the CD trade‐off has been tested for plants across the whole life cycle in a natural multispecies plant community, and more comprehensive studies are still needed to explore the underlying mechanisms and the linkage between the CD trade‐off and community composition.

## 1 INTRODUCTION

A major objective associated with community assemblages is to explain how different species can coexist in a particular habitat given that all organisms require essentially the similar resources. For a long time, the hypothesis that there is a trade‐off between competitive and dispersal abilities in a given species has been considered to be an important mechanism underlying species coexistence (Levins & Culver, 1971; MacArthur & Wilson, 1967; Muller‐Landau, 2010; Rees, 1995; Tilman, 1994) and affecting species spatial distributions in heterogeneous environments (Amarasekare, 2003; Laroche, Jarne, Perrot, & Massol, 2016). This hypothesis assumes that species differ in their abilities to disperse to new habitats and to compete in the same habitat (Kneitel & Chase, 2004). Therefore, coexistence is achieved through the small number of competition‐oriented species in finite habitats because of their lack of dispersal ability, thus leaving space and resources for those dispersing‐oriented species (Leishman & Murray, 2001; Levine & Rees, 2002; Limberger & Wickham, 2011; Rees, 1995).

The competition–dispersal (hereafter CD) trade‐off has been demonstrated in many theoretical studies (Calcagno, Mouquet, Jarne, & David, 2006; Figueiredo & Connolly, 2012; Orrock & Watling, 2010). Initially, the modeling approaches of Levins and Culver (1971) and Tilman (1994) demonstrated that two or many species, respectively, can coexist under the assumption that a trade‐off exists between competitive and dispersal ability. Subsequent models were developed in an attempt to relax the restrictive assumptions, such as instantaneous competitive exclusion (Holmes & Wilson, 1998; Pacala & Rees, 1998) and fully asymmetric competition (Calcagno et al., 2006; Levine & Rees, 2002), or add demographic stochasticity (Orrock & Watling, 2010) and asymmetric dispersal (Figueiredo & Connolly, 2012). Although the results among models have not yet been completely reconciled, it is clear that CD trade‐offs are likely to play an important role in determining species coexistence.

Despite recent developments in theoretical studies, empirical evidence for the CD trade‐off has led to conflicting results for both animal and plant species. For animals, documented evidence has been obtained in studies of birds (Rodríguez, Jansson, & Andrén, 2007), laboratory cultures of aquatic microfauna (Cadotte, 2006, 2007), an acacia‐ant guild (Stanton, Palmer, & Young, 2002), and artificial microcosms of bruchid beetles (Hunt & Bonsall, 2009) but not in studies of benthic ciliates (Limberger & Wickham, 2011), insect herbivores (Harrison, Thomas, & Lewinsohn, 1995), or parasitoids (Amarasekare, 2000). For plants, most evidence of a CD trade‐off was obtained from indirect tests of the trade‐off between seed size and seed number (hereafter SS trade‐off), which indicates that species with small seeds are more fecund than species with larger seeds (Coomes & Grubb, 2003; Jakobsson & Eriksson, 2000; McEuen & Curran, 2004; Turnbull, Rees, & Crawley, 1999) and can disperse over greater distances (Clark, Macklin, & Wood, 1998; Tamme et al., 2013), while a large seed size is positively associated with seedling survival (Coomes & Grubb, 2003; Walters & Reich, 2000; Westoby, Leishman, Lord, Poorter, & Schoen, 1996) and higher competitive ability in the recruitment phase (Gomes et al., 2018; Moles & Westoby, 2004) but a smaller dispersal distance. So far, the only direct evidence for a CD trade‐off in plants was from a study on Asteraceae in an artificial community in which a trade‐off was found between dispersal ability at the offspring level and competitive ability in the recruitment phase (Jakobsson & Eriksson, 2003).

The lack of experimental evidence for a CD trade‐off may be observed largely because the direct measurement of competitive and dispersal ability is a “logistical nightmare” for most organisms (Amarasekare, 2003; Goldberg & Landa, 1991). Therefore, empirical studies have used numerous proxies for competitive and dispersal ability (Kneitel & Chase, 2004; Limberger & Wickham, 2011), and most have focused on the SS trade‐off. In fact, the trade‐off between seed number and seed size occurs within the reproductive phase, but the trade‐off between competitive and dispersal ability actually occurs over the whole life history (e.g., allocation to reproduction vs. growth; Kneitel & Chase, 2004; Leishman, 2001). So far, to the best of our knowledge, there has been no study testing the CD trade‐off at the life‐history level in natural multispecies plant communities. In this study, we quantified the competitive and dispersal abilities of different plant species using plant traits as proxies. The hypothesis of a CD trade‐off was tested using multispecies natural plant communities in a temperate alpine grassland. Specifically, two related questions were addressed: (a) Does a CD trade‐off exist in alpine grassland communities? and (b) What are the potential mechanisms underlying this pattern?

## 2 MATERIALS AND METHODS

### 2.1 Study site

The study was conducted in Bayanbulak alpine grassland (42°18′N–43°34′N, 82°27′E–86°17′E), located in Hejing County in the Bayingolin Mongol Autonomous Prefecture of Xinjiang Uygur Autonomous Region (referred to as Xinjiang), China. This grassland is in the southern Tianshan Mountains basin, with a mean altitude of 2,500 m, and covers a total area of approximately 23,000 km^2^. This region is the largest stock‐raising base in Xinjiang and one of the most extensive, highly productive pasturelands in China and is also regarded as one of the biodiversity hotspots in central Asia (Zhang et al., 2002). The mean annual temperature in the study area is −4.8°C, ranging from −27.4°C in January to 11.2°C in July. The mean annual precipitation is 265.7 mm, with most (approximately 78.1%) falling during the growing season (from May to August; Li et al., 2015).

### 2.2 Experimental design

**Figure 1:**
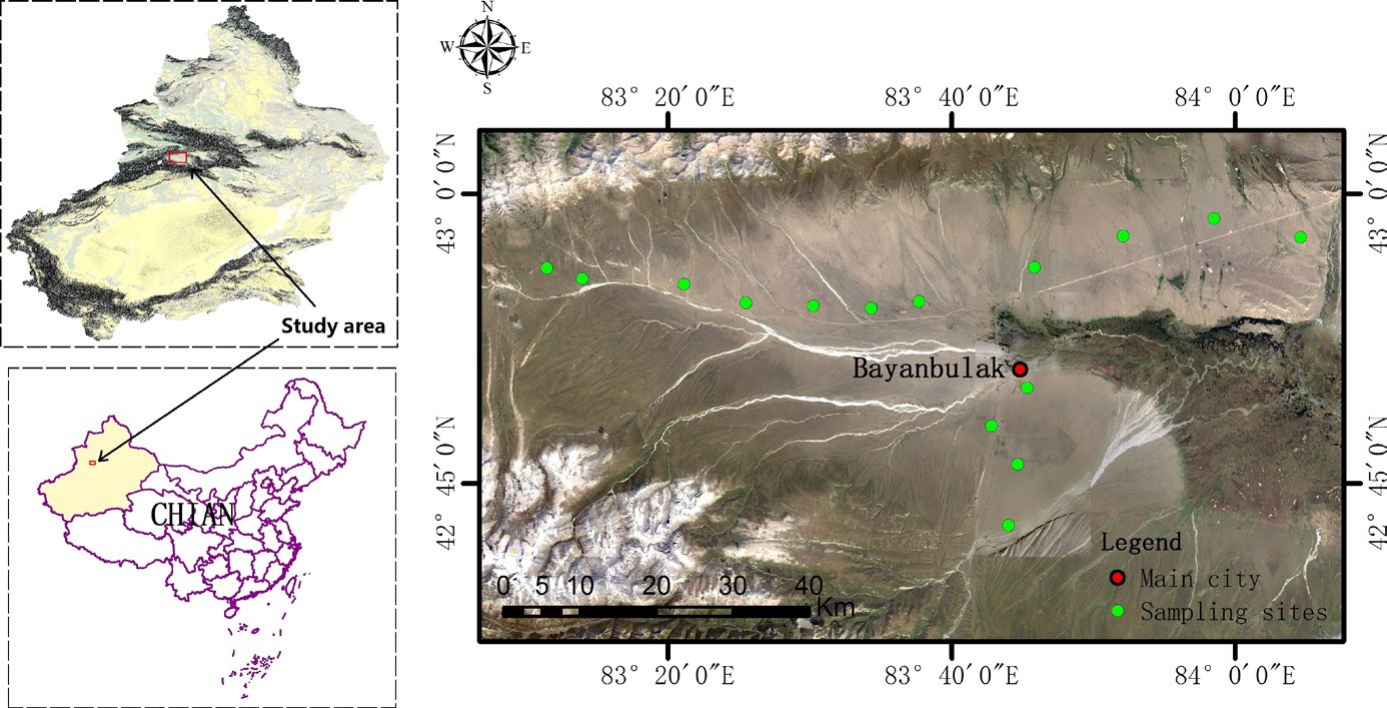
The location of our study area and the distribution of the sampling sites along the transect

From August to October in 2017, 15 sites were regularly sampled along a 98‐km‐length transect (Figure 1). All sampling sites were located near the main roads in this area because the pasturelands were fenced by iron wire fencing. To prevent the roads from disturbing sampling, all sampling sites were established at least 1 km from the roads.

### 2.3 Plant traits

In this study, 47 common species (Supporting Information Appendix S1) were chosen to determine four plant traits: seed mass, ramet dry mass, height, and dispersal mode. Three plant traits were measured in the field (seed mass, ramet dry mass, and height), and dispersal mode was obtained from the literature. The methods of plant trait measurement followed Pérez‐Harguindeguy et al. (2013).

#### 2.3.1 Seed mass

Approximately 500 mature seeds were collected for each species from at least 15 different individuals at the study site. The collected seeds were then air dried, and three subsamples (100 seeds per subsample) were weighed for each species to measure the seed mass (g/100 seeds).

#### 2.3.2 Ramet dry mass and height

For each species, nine undamaged ramets (equivalent to tillers in graminoids and rosettes or root branches in forbs) were randomly collected. The ramet height (cm) was measured from the base to the highest point of the plant. The aboveground parts (stem and leaves) of the measured ramets were then clipped, oven dried at 75°C, and weighed with an accuracy of 10^−4^ g in the laboratory.

#### 2.3.3 Life form

In the method used by Tamme et al. (2013), the life forms were divided into three groups: trees, shrubs, and herbs. In our study, all species were herbs.

#### 2.3.4 Dispersal mode

The dispersal modes of the studied species were divided into four groups in accordance with the morphological features of the seeds: wind dispersal without special mechanisms (i.e., the seeds do not have any special structures to enhance dispersal and are usually dispersed by abiotic factors), wind dispersal with special mechanisms (e.g., plumes, wings), animal dispersal (endo‐, epi‐, and synzoochory by vertebrates, including humans), and ballistic dispersal (Tamme et al., 2013).

### 2.4 Assessment of competitive ability and dispersal ability

We chose the ramet dry mass as a proxy for the competitive ability of a species in our study, in accordance with previous studies (Gaudet & Keddy, 1988; Mahmoud & Grime, 1976; Saeki, Tuda, & Crowley, 2014). The reason for choosing ramet mass as a proxy for competitive ability in our study was that (a) all species were herbs, (b) the aboveground parts of the studied species were almost completely removed by regular grazing during the long winter, and therefore ramet dry mass could represent the amounts of resources a species obtained during the growing season, and (c) our previous study also suggested that larger species have advantages in competition, being more abundant than smaller species in alpine pastures (Supporting Information Appendix S2).

Many studies have documented that dispersal ability is strongly related to some plant traits (Thomson, Moles, Auld, & Kingsford, 2011; Vittoz & Engler, 2007; Willson, 1993). For example, Tamme et al. (2013) constructed five models based on cross‐validation techniques and global datasets to measure the predictive power of simple plant traits in estimating the maximum dispersal distance of plants. In this study, we used the dispeRsal() function (argument model = 2) provided by Tamme et al. (2013) to quantify dispersal ability based on four traits (seed mass, plant height, life form, and dispersal mode).

### 2.5 Data analysis

All species in our study were divided into two functional groups: graminoids and forbs. First, the dispersal ability ranks were quantified using the dispeRsal() function provided by Tamme et al. (2013). Meanwhile, we ranked the mean values of the ramet dry mass to obtain the competitive ability ranks for our target species. In our study, we used the rank data to represent the dispersal and competitive ability of each species rather than the raw data because rank data are more suitable for the analysis and plotting.

Next, we used simple linear regression to test the relationships between the ranks of the competitive and dispersal ability of all species, graminoids and forbs. Then, a phylogenetic tree of the studied species was built using Phylomatic and Phylocom based on the published phylogenetic supertree of angiosperm families and APG III (Webb, Ackerly, & Kembel, 2008; Webb & Donoghue, 2005), and a phylogenetically independent contrasts (PICs) analysis was performed to test the potential effects of the phylogenetic relationships among these species on the correlations between competitive and dispersal ability (Felsenstein, 1985; Webb, Ackerly, McPeek, & Donoghue, 2002).

Lastly, we counted the number of species undergo clonal reproduction and nonclonal reproduction in graminoids and forbs, respectively. The ratio of clonal reproduction species in graminoids and forbs was also calculated.

All statistical analyses were performed in R (R Development Core Team, 2013), and the PICs were analyzed using the multi2di() and pic() functions in the R packages ape and ade4 (Dray & Dufour, 2007; Paradis, Claude, & Strimmer, 2004).

## 3 RESULTS

**Figure 2:**
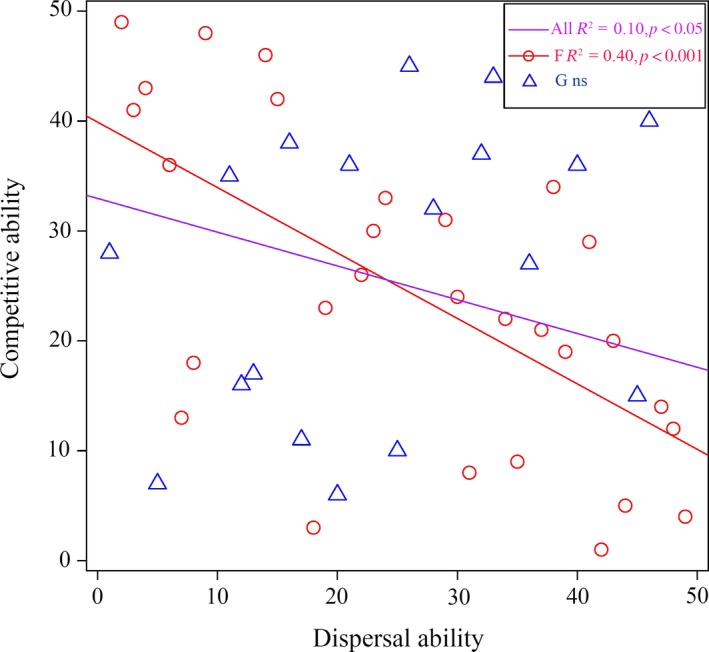
Relationship between the competitive ability ranks and dispersal ability ranks among plant species. *R* ^2^ and *p* values were estimated from simple linear regression. (All: all species; F: forbs; G: graminoids.)

Significant negative relationships between competitive ability and dispersal ability for all species (*R* ^2^ = 0.1023, *p* = 0.0284, Table 1, Figure 2) and for the forbs (*R* ^2^ = 0.4027, *p* = 0.0002, Table 1, Figure 2) were found in our study. But in graminoids, this relationship was not significant (Table 1, Figure 2). In addition, the statistical significance of these relationships did not change after the PICs analysis (Table 1). See Supporting Information Appendix S1 for details on the species list, plant traits (ramet dry mass (g), height (cm), seed mass (g/100 seeds), and dispersal mode), competitive ability and dispersal ability ranks.

**Table 1:** The results of simple regression and simple regression based on PICs (phylogenetically independent contrasts) between competitive ability and dispersal ability

Functional group	Simple regression			Simple regression after PICs		
	Slope	*R* ^2^	*p*	Slope	*R* ^2^	*p*
All species	−0.30	0.1023	**0.0284**	−0.01	0.11	**0.0235**
Graminoids	0.35	0.1183	0.1622	0.1770	0.0253	0.5709
Forbs	−0.60	0.4027	**0.0002**	−0.62	0.4884	**0.0001**

Significant results (*p* < 0.05) are in bold.

In total, 18 graminoid species and 29 forb species that have sexual reproduction were included in our study. In graminoids, 17 species (94.44%) also undergo clonal reproduction in their life history. In contrast, only one species (*Vicia tenuifolia*) undergo clonal reproduction in 29 forb species (Table 2).

**Table 2:** The number of species undergo clonal reproduction and nonclonal reproduction in graminoids and forbs

Functional group	Clonal reproduction	Nonclonal reproduction	Clonal/nonclonal (%)
Graminoids	17	1	94.44
Forbs	1	28	3.57

## 4 DISCUSSION

Our results showed that the CD trade‐off existed for species in the alpine grassland community but that there was a difference between the functional groups of graminoids and forbs. A significant trade‐off between competitive ability and dispersal ability was found for the forbs but not for the graminoids. This result might have occurred because of different resource capture and allocation strategies between the forb species and graminoid species.

Many previous studies have documented the trade‐offs between competitive and dispersal ability due to resource constraints during the life history of organisms (Leishman, 2001; Limberger & Wickham, 2011). For instance, when water, nutrients, and light are strictly limited, some species might allocate more resources to competitive ability to compete for limited resources (Barradas, Caswell, & Cohen, 1996; Tilman, 1988); in contrast, other species might allocate more resources to dispersal ability to increase the probability of colonizing new patches (Baker, 1972). The significantly negative relationship between competitive ability and dispersal ability in forbs suggests that those species are constrained to be either good competitors or good dispersers but not both. This implies that there might be resource constraints for forbs at our study site. Indeed, studies have suggested that the constrained resources in alpine plant communities could include low temperatures and limiting nutrients, such as nitrogen and phosphorus (Billings & Mooney, 1968; Ren et al., 2010; Zhang & Welker, 1996; Zhou et al., 2017). However, more comprehensive studies are needed to distinguish the important roles of those resources in determining the patterns of CD trade‐offs.

In contrast to forbs, the relationship between competitive and dispersal ability in graminoid species was not significant at our study site, suggesting that the CD trade‐off might not exist in those species. A probable explanation is that graminoid species may not be restrictedly constrained by resources, largely because, unlike forbs, which are strongly restricted to sexual reproduction, 94.44% the graminoid species undergo both sexual and clonal reproduction at our study site. The ability to undergo clonal reproduction allows graminoid species to find more benign microenvironments, forage for nutrients across a large area, transport acquired resources among different clone parts and store resources in clonal structures (Evans & Cain, 1995; Klimešová, Martínková, & Ottaviani, 2018; Van groenendael, 1997). In fact, the average ramet mass of graminoids (2.69 ± 1.83 g) is slightly greater than that of forbs (1.69 ± 2.2 g; but not significantly so) at our study site. In a previous study, Venable (1992) emphasized the importance of individual size in CD trade‐offs. In accordance with his model, our results showed that a null correlation between competition and dispersal ability might sometimes occur in graminoid species due to variation in individual size (ramet mass). Interestingly, we observed that some large‐sized graminoids are superior competitors but not inferior colonizers, such as *Leymus secalinus*, *Festuca gigantea, *and *Festuca ovina*.

In this study, we revealed that the CD trade‐off depends on plant traits that are closely related to competitive or dispersal ability. Indeed, previous studies have suggested that relationships among species’ traits related to species’ competitive and dispersal ability play an important role in determining the absence or presence of a CD trade‐off (Pastore et al., 2014; Suding, Goldberg, & Hartman, 2003). Moreover, the CD trade‐off could be highly likely under the conditions of resource constraints, which result in negative correlations between plant traits (Limberger & Wickham, 2011). For example, the trade‐offs between biomass allocation to roots and reproduction in plant species could be finally translated into a trade‐off between the ability to compete for soil nutrients and the ability to colonize abandoned fields (Tilman & Wedin, 1991). Overall, in our study, competitive ability and dispersal ability are both determined by the same trait but in an opposing way (e.g., a large ramet size is related to large seeds and high competitive ability, while large seeds lead to low dispersal ability), leading to the CD trade‐off. Furthermore, the PICs analysis indicated that the phylogenetic effect did not change the observed relationships in our study, suggesting that the CD trade‐off relationship could be weakly influenced by the phylogenetic relatedness among species.

## 5 CONCLUSION

In a natural alpine grassland community, we quantified the competitive and dispersal abilities of different species based on 4 plant functional traits. We found that the CD trade‐off existed in forb species but not in graminoid species, perhaps because graminoid species undergo clonal reproduction and are thus not strictly limited by resource availability. In the future, more comprehensive studies are needed to detect the effects of limiting environmental factors on the patterns of CD trade‐offs and link them to community assembly and species coexistence.

## CONFLICT OF INTEREST

None declared.

## AUTHOR CONTRIBUTIONS

Xiaolong Zhou and Qingdong Shi conceived and designed the experiments. Xiaolong Zhou and Chengzhi Li performed the experiments. Xiaolong Zhou and Honglin Li analyzed the data and wrote the manuscript.

## Data Availability

Data underlying this article can be accessed on Dryad Digital Repository (https://doi.org/10.5061/dryad.3st6vs3) and used under the Creative Commons Attribution license.
