# Supplementary material for: The competition–dispersal trade‐off exists in forbs but not in graminoids: A case study from multispecies alpine grassland communities
Source: Ecol Evol. 2019 Jan 11;9(3):1403–9. doi: 10.1002/ece3.4856 (PMC6374675; doi:10.1002/ece3.4856)
Supplement: Supplementary file 1 [file ECE3-9-1403-s001.docx]

**Appendix 1.** **The name list, plant traits (ramet mass (g), height (cm), seed mass (g), dispersal mode) competitive ability ranks and dispersal ability ranks of the species in our study site. (G:graminoids, F:forbs)**

| **Species** | **G/F** | **Ramet mass** | **Seed mass** | **Height** | **Dispersal mode** | **Competitive ability** | **Dispersal ability** |
| --- | --- | --- | --- | --- | --- | --- | --- |
| *Aconitum gymnandrum* | F | 0.67 | 0.08 | 34.46 | wind.none | 20 | 43 |
| *Agropyron cristatum* | G | 0.51 | 0.08 | 23.25 | wind.special | 11 | 17 |
| *Agropyron pectinatum* | G | 3.21 | 0.03 | 50.95 | wind.special | 35 | 11 |
| *Agrostis gigantea* | G | 5.53 | 0.09 | 100.71 | wind.special | 45 | 26 |
| *Agrostis tenuis* | G | 1.36 | 0.01 | 69.57 | wind.special | 27 | 36 |
| *Allium atrosanguineum* | F | 6.88 | 0.24 | 102.00 | wind.none | 46 | 14 |
| *Allium glomeratum* | F | 0.29 | 0.08 | 21.50 | wind.none | 9 | 35 |
| *Artemisia leucophylla* | F | 0.67 | 0.01 | 26.62 | wind.special | 19 | 39 |
| *Artemisia mongoliea* | F | 2.03 | 0.01 | 34.83 | wind.special | 30 | 23 |
| *Blysmus sinocompressus* | F | 0.66 | 0.01 | 22.90 | wind.none | 7 | 5 |
| *Carex rhynchophysa* | F | 4.71 | 0.06 | 31.43 | wind.none | 6 | 20 |
| *Cerastium fontanum* subsp. *triviale* | F | 0.07 | 0.02 | 13.55 | wind.none | 1 | 42 |
| *Chenopodium glaucum* | F | 0.81 | 0.05 | 13.89 | wind.none | 23 | 19 |
| *Deschampsia caespitosa* | G | 3.78 | 0.03 | 80.59 | wind.special | 37 | 32 |
| *Elymus nutans* | G | 3.31 | 0.31 | 74.08 | animal | 36 | 21 |
| *Euphrasia pectinata* | F | 5.07 | 0.62 | 63.17 | wind.none | 18 | 8 |
| *Festuca gigantea* | G | 4.36 | 0.02 | 53.88 | wind.special | 44 | 33 |
| *Festuca ovina* | G | 5.17 | 0.03 | 37.81 | wind.special | 36 | 40 |
| *Gentiana karelinii* | F | 0.10 | 0.01 | 5.75 | wind.none | 3 | 18 |
| *Geranium pratense* | F | 0.19 | 0.09 | 14.40 | ballistic | 43 | 4 |
| *Halenia elliptica* | F | 0.27 | 0.05 | 27.75 | wind.none | 8 | 31 |
| *Heteropappus altaicus* | F | 0.53 | 0.05 | 13.57 | wind.special | 12 | 48 |
| *Ixeris polycephala* | F | 0.71 | 0.11 | 19.55 | wind.special | 21 | 37 |
| *Koeleria cristata* | G | 0.62 | 0.02 | 49.84 | wind.special | 15 | 45 |
| *Leontopodium nanum* | F | 0.15 | 0.01 | 13.90 | wind.special | 36 | 6 |
| *Leontopodium souliei* | F | 0.54 | 0.01 | 24.62 | wind.special | 13 | 7 |
| *Leymus secalinus* | G | 4.70 | 0.27 | 74.87 | animal | 40 | 46 |
| *Lomatogonium carinthiacum* | F | 0.92 | 0.02 | 26.61 | wind.none | 5 | 44 |
| *Melica altissima* | G | 0.66 | 0.02 | 75.28 | wind.special | 17 | 13 |
| *Microula sikkimensis* | F | 0.84 | 0.14 | 42.25 | animal | 14 | 47 |
| *Pedicularis cheilanthifolia* | F | 0.23 | 0.05 | 20.10 | wind.none | 41 | 3 |
| *Phlomis umbrosa* | F | 8.98 | 0.48 | 49.80 | wind.none | 48 | 9 |
| *Plantago cornuti* | F | 2.83 | 0.02 | 18.58 | wind.none | 34 | 38 |
| *Poa pratensis* | G | 2.51 | 0.04 | 56.10 | wind.special | 32 | 28 |
| *Polygonum songoricum* | F | 0.57 | 0.53 | 33.68 | wind.special | 24 | 30 |
| *Potentilla multifida* | F | 4.87 | 0.04 | 21.26 | wind.none | 42 | 15 |
| *Potentilla soongarica* | F | 2.07 | 0.06 | 23.50 | wind.none | 31 | 29 |
| *Psathyrostachys juncea* | G | 0.63 | 0.10 | 48.98 | wind.special | 16 | 12 |
| *Saposhnikovia divaricata* | F | 2.72 | 0.06 | 48.58 | wind.none | 33 | 24 |
| *Saussurea nigrescens* | F | 1.94 | 0.15 | 38.69 | wind.special | 29 | 41 |
| *Senecio asiaticus* | F | 0.74 | 0.01 | 34.07 | wind.special | 22 | 34 |
| *Setaria viridis* | G | 0.34 | 0.03 | 20.30 | wind.special | 10 | 25 |
| *Silene aprica* | F | 0.13 | 0.02 | 25.10 | wind.none | 4 | 49 |
| *Stipa aliena* | G | 4.17 | 0.44 | 81.76 | animal | 38 | 16 |
| *Stipa purpurea* | G | 1.90 | 0.23 | 32.23 | animal | 28 | 1 |
| *Thalictrum alpinum* | F | 1.18 | 0.13 | 29.53 | wind.none | 26 | 22 |
| *Vicia tenuifolia* | F | 17.09 | 2.40 | 17.33 | ballistic | 49 | 2 |
