# Supplementary material for: The competition–dispersal trade‐off exists in forbs but not in graminoids: A case study from multispecies alpine grassland communities
Source: Ecol Evol. 2019 Jan 11;9(3):1403–9. doi: 10.1002/ece3.4856 (PMC6374675; doi:10.1002/ece3.4856)
Supplement: Supplementary file 2 [file ECE3-9-1403-s002.docx]

**Appendix 2** Relationship between the species relative abundance and ramet mass in common species in a alpine meadow community. R and P value were estimated from Pearson’s correlation test.


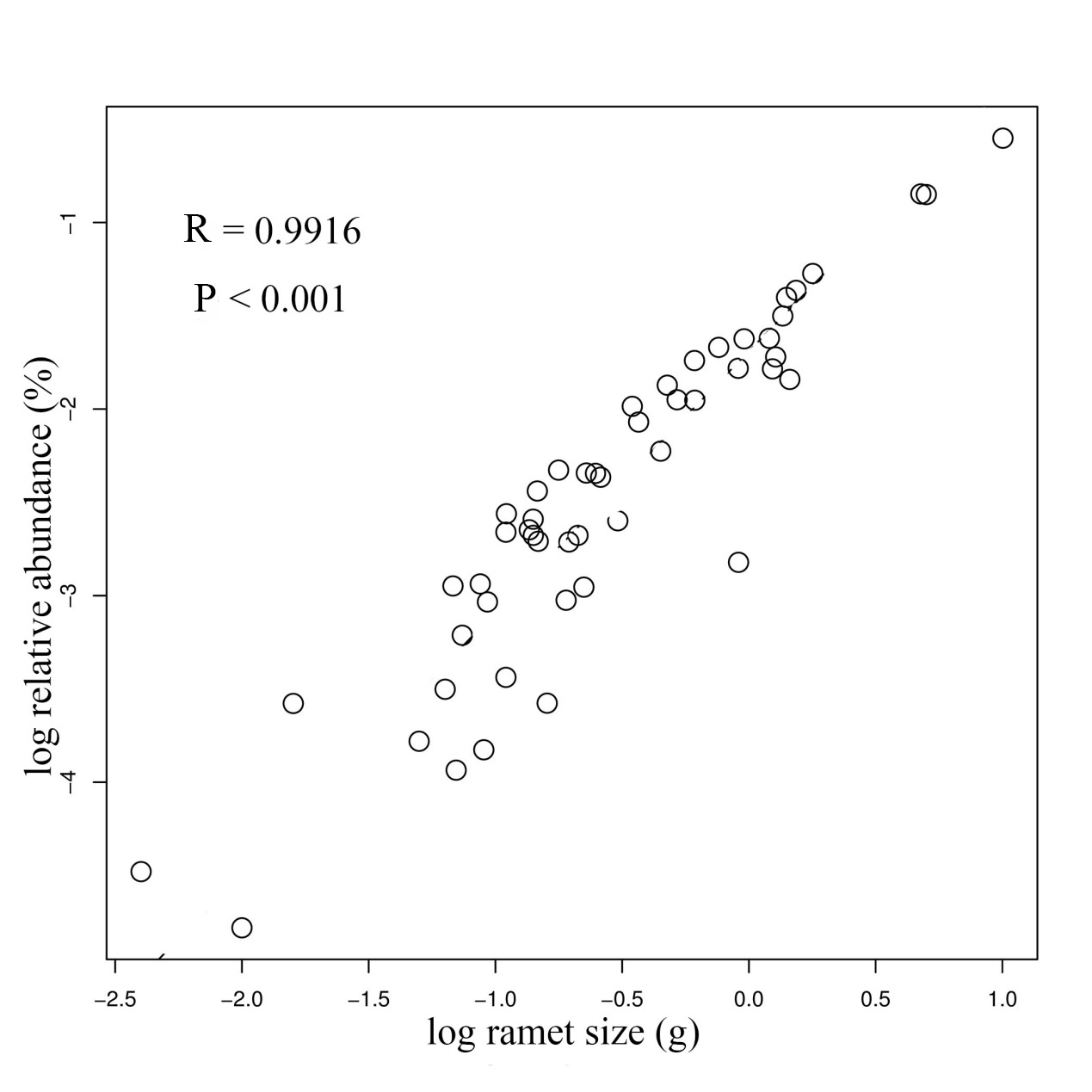


**Methods:**

The experiment was established in April 2011 in an enclosed area of ﬂat alpine meadow, where grazing was only allowed during the nonproductve winter. In late May 2013, 18 5 × 5 m plots, each separated by 30 m, were established in a 3 km × 1 km area of homogeneous meadow. At the middle of August 2013, a 0.5 × 0.5 m quadrat was harvested from each plots. The green, aboveground parts (stems and leaves) were clipped and sorted by species and brought to the laboratory. The green parts were dried at 70°C for 48 hr and weighed (0.01 g) to estmate biomass of each species. In 2013, ramet size were measured in 52 common species following the ﬂowering phase. For each species, we randomly sampled nine fully developed and undamaged ramets.
